# Serum phosphate and social deprivation independently predict all-cause mortality in chronic kidney disease

**DOI:** 10.1186/s12882-015-0187-1

**Published:** 2015-12-01

**Authors:** Marit D. Solbu, Peter C. Thomson, Sarah Macpherson, Mark D. Findlay, Kathryn K Stevens, Rajan K. Patel, Sandosh Padmanabhan, Alan G Jardine, Patrick B. Mark

**Affiliations:** Institute of Cardiovascular and Medical Sciences, University of Glasgow, 126 University Place, Glasgow, G12 8TA UK; Section of Nephrology, University Hospital of North Norway, N-9038 Tromsø, Norway; Glasgow Renal & Transplant Unit, The Queen Elizabeth University Hospital, Glasgow, 1345 Govan Road, Glasgow, G51 4TF UK

**Keywords:** Chronic kidney disease, Mortality, Multiple deprivation, Phosphate, Renal replacement therapy

## Abstract

**Background:**

Hyperphosphataemia is linked to cardiovascular disease and mortality in chronic kidney disease (CKD). Outcome in CKD is also affected by socioeconomic status. The objective of this study was to assess the associations between serum phosphate, multiple deprivation and outcome in CKD patients.

**Methods:**

All adult patients currently not on renal replacement therapy (RRT), with first time attendance to the renal outpatient clinics in the Glasgow area between July 2010 and June 2014, were included in this prospective study. Area socioeconomic status was assessed as quintiles of the Scottish Index of Multiple Deprivation (SIMD). Outcomes were all-cause and cardiovascular mortality and commencement of RRT.

**Results:**

The cohort included 2950 patients with a median (interquartile range) age 67.6 (53.6–76.9) years. Median (interquartile range) eGFR was 38.1 (26.3–63.5) ml/min/1.73 m^2^, mean (±standard deviation) phosphate was 1.13 (±0.24) mmol/L and 31.6 % belonged to the most deprived quintile (SIMD quintile I). During follow-up 375 patients died and 98 commenced RRT. Phosphate ≥1.50 mmol/L was associated with all-cause (hazard ratio (HR) 2.51; 95 % confidence interval (CI) 1.63-3.89) and cardiovascular (HR 5.05; 95 % CI 1.90–13.46) mortality when compared to phosphate 0.90–1.09 mmol/L in multivariable analyses. SIMD quintile I was independently associated with all-cause mortality. Phosphate did not weaken the association between deprivation index and mortality, and there was no interaction between phosphate and SIMD quintiles. Neither phosphate nor SIMD predicted commencement of RRT.

**Conclusions:**

Multiple deprivation and serum phosphate were strong, independent predictors of all-cause mortality in CKD and showed no interaction. Phosphate also predicted cardiovascular mortality. The results suggest that phosphate lowering should be pursued regardless of socioeconomic status.

## Background

Disorders of mineral metabolism, in particular hyperphosphataemia, are linked to cardiovascular morbidity and mortality in chronic kidney disease (CKD) [1]. Independent associations between serum phosphate and cardiovascular and all-cause mortality have been shown among persons on chronic dialysis [2, 3] and in non-dialysis CKD patients [4–6]. The proposed mechanisms for this association involve endothelial dysfunction [7] and vascular calcification [8–10].

The burden of CKD differs substantially across socioeconomic groups, with higher CKD incidence and prevalence rates in lower compared to higher socioeconomic groups [11–16]. Moreover, the risk of adverse outcome in CKD, such as CKD progression [17, 18] and mortality [19, 20], is increased in patients with lower socioeconomic status. These associations are only partly explained by the high rates of traditional cardiovascular and renal risk factors observed in these groups [21–23]. Factors characterising deprivation and thus having an unfavourable impact on health outcome [24] may vary between geographical regions, and these associations should be examined in various areas.

Serum phosphate has been shown to increase with decreasing socioeconomic status independent of estimated glomerular filtration rate (eGFR) in the general population [25] and in CKD patients [26]. Dietary differences with a high intake of processed food (“fast food”) containing easily absorbable phosphorus additives in deprived groups may explain this association [27, 28]*.*

It is not known whether the higher phosphate level partially can explain the association between low socioeconomic status and adverse outcome in CKD.

The objective of the present prospective study was to assess the associations between serum phosphate, multiple deprivation and CKD outcome in a Scottish cohort of CKD patients not requiring renal replacement therapy (RRT) at baseline. Specifically, we wanted to address whether hyperphosphataemia partly could explain a more adverse outcome among patients with low socioeconomic status.

## Subjects and Methods

### Study population

The Glasgow Renal and Transplant Unit serves a population of 1.5 million people across West and Central Scotland. All adult patients attending the outpatient clinics for the first time between 1^st^ July 2010 and 30^th^ June 2014 were identified through the Strathclyde Electronic Renal Patient Record (Vitalpulse, UK) (*n* = 3676). Patients with previous kidney transplants or on chronic dialysis (*n* = 170) were excluded. Also, patients with missing serum phosphate values (*n* = 556) were excluded from the present study. Use of anonymised data from this database has been approved by the West of Scotland Ethics Committee for use of NHS Greater Glasgow and Clyde ‘Safe Haven’ data for research.

### Measurements

For each patient, demographic and clinical data were recorded on their first attendance to the outpatient clinic. Previous cardiovascular disease and known diabetes were assessed from baseline data recorded in the electronic patient record, derived from the General Practitioner records for each patient. Laboratory data were acquired from the local hospital laboratories. Height and weight were measured and blood pressure was recorded by the nurses at each location. Due to a large number of missing values for height, we applied body weight (in kg) instead of body mass index as a measure of obesity in the multivariable analyses. Creatinine based eGFR was calculated using the Chronic Kidney Disease Epidemiology Collaboration (CKD-EPI) equation [29].

Socioeconomic status for each patient was obtained using the Scottish Index of multiple Deprivation (SIMD) system. SIMD is the Scottish Government’s widely accepted tool for identifying areas suffering from deprivation [30]. Scotland is divided into 6505 so-called datazones, each datazone including an average of 800 persons. SIMD combines 38 indicators across 7 domains (employment, income, health, education, geographic access to service, crime and housing), and each domain is weighted according to its importance to the overall concept of multiple deprivation [30, 31]. The SIMD for all datazones are ranked from 1 (most deprived) to 6505 (least deprived), resulting in a relative measure of area deprivation. The SIMD rank for each patient was acquired by linking the patient’s postcode to a SIMD datazone. In the present paper, SIMD quintiles recorded from the central SIMD database were applied. Quintile I comprise persons living in the most deprived areas.

### Outcomes

Date of death was received from the electronic patient record which is cross referenced with the General Register for Scotland. Cause of death was determined from death certificates if available in the patient record, or adjudicated by the authors when the electronic patient record contained information about the final course leading to death. A death was assigned a cardiovascular cause if the patient died from any cardiac disease, stroke or peripheral vascular disease. Date of commencing RRT was retrieved from the electronic patient record. When assessing cardiovascular mortality and RRT commencement, patients were censored on the date of death if they did not reach the end-point of interest, but died from other or unknown cause. Follow-up time was assigned from the first renal clinic attendance date to the date of death or 3^rd^ October 2014 for all-cause and cardiovascular mortality, and to the date of commencing RRT, the date of death or 3^rd^ October 2014, whichever came first, for the RRT endpoint.

### Statistical analyses

Data are presented as mean (±standard deviation [SD]), median (interquartile range) or number (percentage) as appropriate. Serum phosphate was categorised into five groups: <0.90 mmol/L, 0.90-1.09 mmol/L, 1.10–1.29 mmol/L, 1.30–1.49 mmol/L and ≥1.50 mmol/L. Differences in baseline characteristics across these phosphate categories were assessed using one-way ANOVA, Kruskall-Wallis test and Jonckheere-Terpstra trend test and Chi square test as appropriate, and P for linear trend was reported. Similarly, differences in characteristics across SIMD quintile groups were examined. In these analyses, SIMD quintiles II and III were merged into one group, as were quintiles IV and V.

We used multivariable linear regression analysis to assess the cross-sectional association between phosphate and other variables.

Crude incidence rates were calculated as number of events per 1000 person years at risk. Differences in incidence rates across the phosphate categories were tested for by a normal test with continuity correction. Because a trend towards a J-formed association between phosphate and all-cause mortality was observed in multivariable adjusted analyses, the phosphate range 0.90–1.09 mmol/L was set as reference.

Cox proportional hazard regression analyses were used to examine the associations between phosphate, SIMD quintiles and the three endpoints. Covariates were entered block wise. The SIMD quintiles and phosphate categories, respectively, were adjusted for sex, age and eGFR in two separate models, Model 1a and 1b. In Model 2, SIMD quintiles, phosphate categories, age sex and eGFR were entered into the same model. Model 3 tested the addition of traditional cardiovascular risk factors (systolic and diastolic blood pressure, body weight, presence/absence of diabetes and/or coronary heart disease) to Model 2, and in Model 4, urinary protein-creatinine ratio (PCR), serum albumin and haemoglobin were added. Relevant interactions, including interaction between the SIMD quintiles and phosphate for the prediction of each endpoint, were tested for. In separate Cox regression analyses, the cohort was classified into four groups according to their combination of phosphate (≥1.50 mmol/L vs. all others) and SIMD quintile (quintile I vs. all others). The association with all-cause mortality was assessed, including the same covariates as in Model 4 described above. The proportional hazard assumption was checked by visual inspection of the –log-log curves. *P* values <0.05 were considered significant. Analyses were run using IBM SPSS Statistics software version 22 (IBM Corp., Armonk, NY).

## Results

### Baseline characteristics

The 556 patients who had missing phosphate measurements and therefore were excluded from the study, were significantly younger, had lower PCR and less frequently diabetes than the 2950 patients (1475 men and 1475 women) who comprised the study cohort. However, the 556 excluded patients did not differ significantly with regards to other properties including distribution of CKD stages and SIMD quintiles (P for differences between the groups =0.14 and 0.77, respectively). During follow-up, 76 (13.7 %) of patients with missing phosphate measurements died; this was not significantly different from the death rate in the study cohort (12.7 %; P for between-group difference =0.54). The study cohort had median age 67.6 (53.6–76.9) years, median eGFR 38.1 (26.3–63.6) ml/min/1.73 m^2^, mean phosphate 1.13 (±0.24) mmol/L, and 929 (31.6 %) were categorised into the most deprived quintile (SIMD quintile I). CKD stages were distributed as follows: 797 (26.9 %) had stage 1 or 2, 1161 (39.4 %) had stage 3, 846 (28.7 %) stage 4 and 147 patients (5.0 %) had stage 5 CKD. Fifty-two (1.8 %) persons attended for live donor assessment.

Baseline characteristics were assessed according to the phosphate categories. Compared to the category with phosphate 0.90–1.09 mmol/L (*n* = 912), the high phosphate category (phosphate ≥1.50 mmol/L; *n* = 177) was characterised by more women (58.2 % vs. 44.8 %) and a higher percentage of persons residing in the lowest SIMD quintile areas (33.3 % vs. 30.9 %). As expected, eGFR was lower in the high phosphate category. There was no consistent trend towards a worse cardiovascular risk profile in the high versus low phosphate category.

Characteristics were compared across the SIMD quintile groups (Table 1). Age was lower and the percentage of women higher in the most deprived compared to the least deprived quintiles. Phosphate was significantly higher in SIMD quintile I than in quintiles IV and V, whereas eGFR did not differ significantly across the SIMD quintiles. Diabetes was more frequent in the most deprived quintile, whereas coronary heart disease was evenly distributed across the SIMD quintiles.

**Table 1:** Baseline characteristics according to Scottish Index of Multiple Deprivation (SIMD) quintiles 2012

	SIMD quintile I (*n* = 929)		SIMD quintile II–III (*n* = 1096)		SIMD quintile IV–V (*n* = 912)		P for linear trend
Men, n (%)	443	(47.7)	538	(49.1)	486	(53.3)	0.016
Age, years	66.3	(51.0–76.2)	68.2	(54.5–77.4)	68.2	(54.6–77.3)	0.016
Phosphate, mmol/L	1.15	±0.24	1.15	±0.25	1.11	±0.24	0.001
Phosphate >1.30 mmol/L	207	(22.3)	237	(21.6)	165	(18.1)	0.002
eGFR, mL/min/1.73 m^2^	39.9	(27.3–67.8)	36.0	(25.5–59.0)	39.3	(27.0–63.7)	0.7
Urinary protein-creatinine ratio, mg/mmol	32.6	(7.6–137.8)	37.0	(9.2–130.1)	25.0	(5.6–102.6)	0.041
Albumin, g/L	36.2	±5.7	37.3	±5.8	37.5	±5.8	<0.001
Haemoglobin, g/L	123.3	±21.4	123.4	±20.7	124.9	±20.3	0.11
Systolic blood pressure, mm Hg	145.9	±25.1	148.5	±26.1	148.5	±25.8	0.035
Diastolic blood pressure, mm Hg	77.9	±13.0	77.7	±13.4	78.3	±12.9	0.48
Body weight, kg	80.7	±21.4	80.8	±19.8	80.0	±19.4	0.46
Body mass index, kg/m^2^	30.0	±7.3	29.6	±6.3	28.6	±6.4	<0.001
Diabetes, n (%)	301	(32.4)	352	(32.1)	229	(25.1)	0.001
Coronary heart disease, n (%)	162	(17.4)	196	(17.9)	143	(15.7)	0.32

Values are given as mean ± SD, median (interquartile range) or number (percentage) as appropriate. *eGFR* Estimated glomerular filtration rate according to the CKD-EPI equation, *SIMD* Scottish index of multiple deprivation. Quintile I comprise the most deprived and quintile V the least deprived

### Predictors of serum phosphate

Table 2 displays the variables that were independently associated with serum phosphate. In addition to socioeconomic deprivation, lower age, eGFR and body weight, increasing urinary PCR and female sex were among the predictors of increasing phosphate.

**Table 2:** Predictors of serum phosphate (mmol/L) at baseline in multivariable linear regression

		β coefficient	95 % confidence interval	Standardised β coefficient	*P* value
Sex, male		−0.35	(−0.53 - -0.17)	−0.077	<0.001
Age, per year		−0.003	(−0.003 - -0.002)	−0.207	<0.001
eGFR, per ml/min/1.73 m^2^		−0.001	(−0.002 - -0.001)	−0.177	<0.001
SIMD quintiles	I	Ref.			
	II–III	−0.011	(−0.003 - 0.010)	−0.021	0.31
	IV–V	−0.029	(−0.049 - -0.009)	−0.058	0.005
Diastolic blood pressure, per mm Hg		-0.001	(-0.002 - 0.000)	-0.047	0.053
Body weight, per kg		−0.001	(−0.002 - -0.001)	−0.111	<0.001
Urinary PCR, per 10 mg/mmol		0.00078	(0.00050 - 0.00107)	0.113	<0.001
Haemoglobin, per g/L		−0.002	(−0.003 - -0.001)	−0.174	<0.001
Potassium, per mmol/L		0.073	(0.057 - 0.090)	0.173	<0.001
Adjusted R^2^	0.172				

*eGFR* Estimated glomerular filtration rate, *SIMD* Scottish index of multiple deprivation. Quintile I comprise the most deprived. *PCR* Protein-creatinine ratio. Also adjusted for systolic blood pressure, albumin adjusted serum calcium, serum albumin, presence/absence of diabetes and/or coronary heart disease

### Serum phosphate, deprivation and outcomes

Median follow-up time was 1.71 (0.93–2.79) years for all-cause and cardiovascular mortality, and 1.66 (0.89–2.74) years for RRT commencement. During follow-up, 375 of the patient died. The cause of death was determined in 254 (67.7 %) cases, and among these, 67 patients (26.3 %) died from cardiovascular disease. Ninety eight patients started RRT (80 started in-centre haemodialysis, 12 started peritoneal dialysis and 7 received a kidney transplant pre-emptively).

Crude all-cause mortality rates and incidence rates of RRT initiation increased with increasing phosphate (Table 3A-C). The number of cardiovascular deaths was low in each phosphate category, and the lowest incidence rate was found among patients with phosphate 1.30–1.49 mmol/L. The crude all-cause and cardiovascular mortality rates increased with increasing deprivation. Also, the crude incidence rate for RRT was highest in the most deprived quintile (data not shown).

**Table 3:** Crude incident rates (number of events per 1000 patient years of risk) for all-cause mortality, cardiovascular mortality and commencement of renal replacement therapy according to baseline phosphate categories

A. All-cause mortality							
Phosphate categories	Number of patients at risk	Number of events	Follow-up time (months)		Crude incidence rate	95 % confidence interval	*P* value*
<0.90 mmol/L	422	39	18.89	(10.94–32.46)	50.82	(34.87–66.77)	<0.001
0.90–1.09 mmol/L	912	96	21.90	(11.47–33.66)	54.38	(43.50–65.26)	Ref.
1.10–1.29 mmol/L	1004	130	21.68	(11.37–34.29)	67.48	(55.88–79.08)	<0.001
1.30–1.49 mmol/L	435	64	20.30	(11.63–32.23)	78.90	(59.57–98.23)	<0.001
≥1.50 mmol/L	177	46	17.58	(6.85–32.00)	154.94	(110.17–199.72)	<0.001
Total	2950	375	20.57	(11.14–33.48)	67.36	(60.54–74.18)	
B. Cardiovascular mortality							
Phosphate categories	Number of Patients at risk	Number of Events	Follow-up time (months)		Crude Incidence Rate	95 % Confidence Interval	*P* value*
<0.90 mmol/L	422	6	18.89	(10.94–32.46)	7.82	(1.56–14.07)	0.003
0.90–1.09 mmol/L	912	18	21.90	(11.47–33.66)	10.20	(5.49–14.91)	Ref.
1.10–1.29 mmol/L	1004	24	21.68	(11.37–34.29)	12.46	(7.47–17.44)	0.037
1.30–1.49 mmol/L	435	6	20.30	(11.63–32.23)	7.40	(1.48–13.32)	<0.001
≥1.50 mmol/L	177	13	17.58	(6.85–32.00)	43.79	(19.98–67.59)	<0.001
Total	2950	67	20.57	(11.14–33.48)	12.03	(9.15–14.92)	
C. Renal Replacement Therapy							
Phosphate categories	Number of Patients at risk	Number of Events	Follow-up time (months)		Crude Incidence Rate	95 % Confidence Interval	*P* value*
<0.90 mmol/L	422	4	18.79	(10.93–32.36)	5.24	(0.10–10.38)	<0.001
0.90–1.09 mmol/L	912	14	21.65	(11.18–33.63)	8.00	(3.81–12.18)	Ref.
1.10–1.29 mmol/L	1004	21	20.91	(11.07–33.64)	11.05	(6.32–15.78)	0.004
1.30–1.49 mmol/L	435	26	19.42	(11.07–31.27)	33.26	(20.48–46.05)	<0.001
≥1.50 mmol/L	177	33	13.63	(4.78–28.09)	133.96	(88.25–179.67)	<0.001
Total	2950	98	19.88	(10.68–32.92)	18.01	(14.44–21.75)	

*P value for comparison with the reference group. Follow-up time is given as median (interquartile range)

When age, sex and eGFR were adjusted for, the all-cause mortality risk was increased by 275 % (hazard ratio [HR] 2.75; 95 % confidence interval [CI] 1.89–4.00) in the highest phosphate category compared to reference. Similarly, residing in the two lower SIMD quintile areas implied an elevated risk of death of 63 and 69 %, respectively (Table 4, Model 1a and 1b). The estimates for both the SIMD quintiles and phosphate categories were essentially unchanged when these variables were entered into the same model (Model 2). Adding traditional cardiovascular risk factors to the model slightly weakened the association between deprivation and mortality, but not between phosphate and mortality. However, by including the covariates urinary PCR, serum albumin and haemoglobin, the association between phosphate and mortality also was weakened. In the multivariable adjusted analyses (Model 4) there was a borderline significant association between the lowest phosphate category (<0.90 mmol/L) and all-cause mortality.

**Table 4:** Hazard ratios (HR) and 95 % confidence intervals (CI) for all‐cause mortality. Predictors are baseline variables

		Model 1a and b			Model 2			Model 3			Model 4		
		HR	95 % CI	*P* value	HR	95 % CI	*P* value	HR	95 % CI	*P* value	HR	95 % CI	*P* value
SIMD quintiles	V	Ref.			Ref.			Ref.			Ref.		
	IV	1.28	0.86–1.91	0.22	1.33	0.89–1.99	0.16	1.28	0.85–1.92	0.23	1.46	0.94–2.26	0.09
	III	1.31	0.87–1.96	0.19	1.26	0.84–1.89	0.26	1.22	0.81–1.83	0.34	1.19	0.75–1.87	0.46
	II	1.63	1.13–2.35	0.009	1.60	1.11–2.30	0.012	1.50	1.03–2.17	0.033	1.56	1.04–2.34	0.031
	I	1.69	1.19–2.40	0.003	1.68	11.18–2.38	0.004	1.55	1.09–2.21	0.015	1.60	1.10–2.34	0.015
Phosphate groups	<0.90 mmol/L	1.08	0.74–1.57	0.70	1.09	0.75–1.58	0.67	1.12	0.76–1.64	0.57	1.44	0.97–2.15	0.073
	0.90–1.09 mmol/L	Ref.			Ref.			Ref.			Ref.		
	1.10–1.29 mmol/L	1.24	0.95–1.62	0.12	1.22	0.93–1.59	0.15	1.24	0.95–1.63	0.12	1.20	0.89–1.62	0.24
	1.30–1.49 mmol/L	1.39	1.01–1.93	0.045	1.38	0.99–1.92	0.05	1.38	0.98–1.93	0.06	1.03	0.70–1.51	0.88
	≥1.50 mmol/L	2.75	1.89–4.00	<0.001	2.73	1.88–3.99	<0.001	2.86	1.93–4.24	<0.001	2.51	1.63–3.89	<0.001

A strong association was found between phosphate ≥1.50 mmol/L and cardiovascular mortality (Table 5), and the association was not substantially altered by adding SIMD quintiles, traditional and non-traditional risk factors to the Cox model. Deprivation was not a significant predictor of cardiovascular mortality.

**Table 5:** Hazard ratios (HR) and 95 % confidence intervals (CI) for cardiovascular mortality. Predictors are baseline variables

		Model 1a and b			Model 2			Model 3			Model 4		
		HR	95 % CI	*P* value	HR	95 % CI	*P* value	HR	95 % CI	*P* value	HR	95 % CI	*P* value
SIMD quintiles	V	Ref.			Ref.			Ref.			Ref.		
	IV	0.58	0.21-1.61	0.30	0.65	0.23-1.79	0.40	0.65	0.23-1.80	0.41	0.55	0.17-1.81	0.33
	III	0.93	0.38-2.30	0.88	0.87	0.35-2.16	0.77	0.90	0.36-2.22	0.81	0.69	0.24-1.98	0.49
	II	1.04	0.46-2.35	0.93	1.01	0.45-2.30	0.97	0.96	0.42-2.22	0.93	0.95	0.39-2.35	0.92
	I	1.65	0.80-3.40	0.18	1.65	0.80-3.42	0.18	1.38	0.66-2.90	0.39	1.43	0.66-3.11	0.37
Phosphate groups	<0.90 mmol/L	0.90	0.36-2.28	0.83	0.89	0.35-2.26	0.81	1.11	0.43-2.87	0.83	1.48	0.56-3.94	0.44
	0.90–1.09 mmol/L	Ref.			Ref.			Ref.			Ref.		
	1.10–1.29mmol/L	1.25	0.67–2.32	0.48	1.24	0.67–2.30	0.50	1.50	0.79–2.84	0.22	1.63	0.80–3.31	0.18
	1.30–1.49mmol/L	0.76	0.29–1.94	0.56	0.75	0.29–1.93	0.55	0.97	0.37–2.53	0.95	0.84	0.29–2.44	0.75
	≥1.50 mmol/L	4.75	2.16–10.44	<0.001	4.55	2.07–10.03	<0.001	6.18	2.61–14.65	<0.001	5.05	1.90–13.46	0.001

SIMD quintiles were not associated with RRT initiation in adjusted analyses. High phosphate was associated with commencement of RRT in analyses adjusted for age, sex, eGFR, deprivation and traditional cardiovascular risk factors, but no significant association remained after adjustment for urinary PCR, serum albumin and haemoglobin (Table 6).

**Table 6:** Hazard ratios (HR) and 95 % confidence intervals (CI) for renal replacement therapy. Predictors are baseline variables

		Model 1a and b			Model 2			Model 3			Model 4		
		HR	95 % CI	P value	HR	95 % CI	P value	HR	95 % CI	P value	HR	95 % CI	P value
SIMD quintiles	V	Ref.			Ref.			Ref.			Ref.		
	IV	1.03	0.51-2.05	0.94	1.04	0.52–2.09	0.90	1.02	0.51–2.06	0.95	1.47	0.68–3.19	0.33
	III	0.81	0.38-1.70	0.58	0.83	0.39–1.75	0.62	0.84	0.40–1.79	0.66	1.03	0.45–2.37	0.94
	II	0.44	0.21-0.95	0.037	0.43	0.20–0.93	0.032	0.47	0.22–1.00	0.051	0.47	0.20–1.12	0.087
	I	1.16	0.63-2.10	0.64	1.18	0.65–2.16	0.59	1.12	0.60–20.08	0.73	1.01	0.51–2.01	0.98
Phosphate groups	<0.90 mmol/L	0.77	0.25–2.34	0.64	0.82	0.27–2.52	0.73	1.02	0.33–3.16	0.97	0.95	0.26–3.46	0.94
	0.90–1.09 mmol/L	Ref.			Ref.			Ref.			Ref.		
	1.10–1.29 mmol/L	1.16	0.58–2.29	0.68	1.22	0.61–2.41	0.57	1.37	0.68–2.75	0.38	1.13	0.53–2.43	0.76
	1.30–1.49 mmol/L	1.83	0.94–3.58	0.077	1.76	0.90–3.46	0.098	2.12	1.06–4.24	0.034	1.53	0.72–3.27	0.76
	≥1.50 mmol/L	2.29	1.12–4.71	0.024	2.51	1.24–5.10	0.011	3.03	1.43-6.42	0.004	1.64	0.71-3.82	0.25

*eGFR*, Estimated glomerular filtration rate according to the CKD‐EPI equation, *SIMD* Scottish index of multiple deprivation. Quintile I comprise the most deprived and quintile V the least deprived. *PCR* Protein‐creatinine ratio, *Model 1a* SIMD quintiles, *eGFR* Age and sex, *Model 1b* Phosphate groups, *eGFR* Age and sex, *Model 2* SIMD quintiles, phosphate groups, *eGFR* Age and sex, *Model 3* Model 2 plus traditional cardiovascular risk factors (presence of diabetes and/or coronary heart disease, systolic and diastolic blood pressure, body weight), *Model 4* Model 3 plus non‐traditional risk factors (urinary PCR, serum albumin, haemoglobin)

As a continuous variable serum phosphate was associated with all-cause mortality when adjusted for age, sex, eGFR, SIMD quintiles and traditional cardiovascular risk factors (HR 2.56; 95 % CI 1.72–3.81; *P* < 0.001 per mmol/L increase), but the significant association disappeared with adjustment for urinary PCR, serum albumin and haemoglobin. Similar relationships were seen between serum phosphate and the two other endpoints (data not shown).

There was no significant interaction between serum phosphate and the SIMD quintiles for the prediction of any endpoint. Nor was there any significant interaction between sex and phosphate or between eGFR and phosphate.

**Fig. 1:**
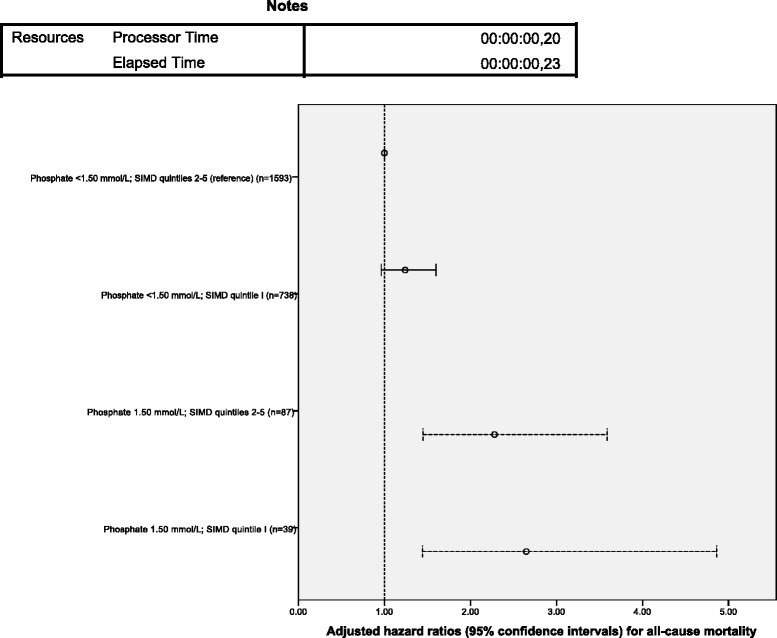
Multivariable adjusted hazard ratios (95 % confidence intervals) for all-cause mortality by categorisation of serum phosphate (<1.50 mmol/L or ≥1.50 mmol/L) and quintiles of Scottish Index of multiple Deprivation (SIMD) at baseline. SIMD quintile 1 comprises the most deprived

**Fig. 2:**
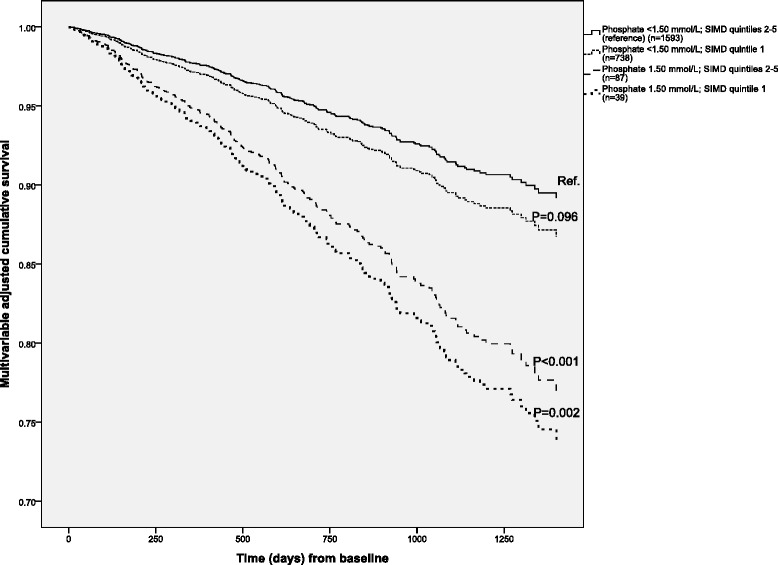
Multivariable adjusted cumulative survival by categorisation of serum phosphate (<1.50 mmol/L or ≥1.50 mmol/L) and quintiles of Scottish Index of multiple Deprivation (SIMD) at baseline. SIMD quintile 1 comprises the most deprived

Figure 1 displays HR (95 % CI) for all-cause mortality when the cohort was classified by the combination of phosphate level and SIMD quintiles, and Fig. 2 shows the corresponding adjusted survival curves. Phosphate ≥1.50 mmol/L was a predictor of death irrespective of SIMD quintile, and the most deprived SIMD quintile was only borderline significantly associated with mortality in this model.

## Discussion

In this large cohort of CKD patients not established on RRT, we found that higher phosphate was an independent predictor of all-cause and cardiovascular mortality. Multiple deprivation, measured as SIMD quintiles, was also independently associated with all-cause mortality. Serum phosphate was significantly higher in patients from the most deprived compared to the least deprived areas, but the association between phosphate and mortality was not altered by deprivation, and there was no interaction between these two variables for the prediction of any endpoint. Therefore, the higher phosphate level observed in patients from the most deprived areas could not explain the increased mortality risk in the deprived group. Nor could the increased mortality risk implied by higher phosphate be explained in terms of socioeconomic deprivation. These associations have never previously been demonstrated in a cohort of CKD patients.

Our data support previous findings of a relationship between slightly increased phosphate and increased risk of all-cause [1, 4–6, 32] and cardiovascular [1, 5, 6] mortality in non-RRT CKD patients.

It has long been recognised that CKD incidence and outcome vary between patients from different socioeconomic groups [12, 17, 18, 20, 23], but the reasons for this variation have not been fully explained [24]. Genetic and other biological aspects, differences in life style, health related behaviour and environmental hazards, and inequalities in health services may be involved. The influence of each factor is believed to differ from one area to another [12]. In a study involving the same Scottish population, a higher incidence of biopsy-proven kidney disease, particularly IgA nephropathy, was observed among patients from areas with lower SIMD ranks compared to patients living in less deprived areas [33]. Consistent with this, a large proportion of the patients in our cohort, 31.6 %, resided in areas ranked in the lowest SIMD quintile.

Individual and area-level socioeconomic disadvantage increase the risk of consuming a disproportionally large amount of inexpensive, unhealthy “fast food” [34, 35]. In addition to their high content of fat, energy and salt, a recent concern regarding processed foods is their high content of easily absorbable inorganic phosphate additives [27]. An association between estimated consumption of phosphate and all-cause and cardiovascular mortality was demonstrated in a sample of healthy US adults with normal eGFR [36]. Persons who consumed high amounts of phosphate were younger, more physically active, better educated and less often classified as poor than the quartile with the lowest phosphate intake. However, the authors discuss the possibility that phosphorus intake may have been systematically underestimated from nutrient databases not updated on phosphate additives in processed foods [36]. In the present study, serum phosphate was significantly higher in SIMD quintile I than in quintiles IV and V. The groups had similar eGFR, and it is reasonable to believe that differences in phosphate intake caused the difference in serum phosphate observed between these groups. Other possible explanations for the higher phosphate relate to the variables found to be independently associated with phosphate (Table 2) and also overrepresented in the most deprived group, including female sex, lower age and higher urinary PCR. However, the lack of interaction between serum phosphate and deprivation in our study does not support a differential effect of phosphate on clinical events according to socioeconomic status, and restriction of phosphate intake should be pursued in CKD patients regardless of socioeconomic status.

The high prevalence of traditional risk factors, such as hypertension, diabetes [21, 37], smoking [22], lack of physical activity [23] and abdominal obesity [23, 38] in lower socioeconomic status groups, partly explains the association between deprivation and adverse outcome in CKD. In our cohort, the patients residing in deprived areas had slightly higher body mass index and the highest prevalence of diabetes. Also, urinary PCR was higher in the lowest SIMD quintile. However, other traditional cardiovascular risk factors were not associated with the lower SIMD quintile. Moreover, low SIMD rank remained an independent significant predictor of mortality, and other explanations should be sought. Unfortunately, information about smoking habits, possibly an important confounder, was not available in our database.

Although phosphate was an independent predictor of RRT commencement when adjusted for eGFR and cardiovascular risk factors, the association did not remain significant when other markers of uraemia including serum albumin were added to the statistical model. An association between serum phosphate and eGFR decline in advanced [39] and earlier [40] stages of CKD has been reported, and increased phosphate has been implicated as a predictor of kidney failure defined as the need for RRT [41, 42]. The discrepancy between these previous reports and the lack of association observed in the present study may be explained in terms of differences in cohorts, observation time and covariate adjustment. Moreover, timing of RRT is a decision based upon evaluation of several laboratory values and symptoms, and practice patterns vary around the world [43]. Multiple deprivation was not independently associated with RRT commencement in the present study.

Our study had several strengths and limitations. The large cohort size and the completeness of all-cause mortality and RRT commencement registration were obvious strengths. The SIMD classification system is a validated, robust indicator of deprivation at area level. In this study, the index was used as a proxy for individual deprivation. However, there may be deprived individuals living in less deprived areas and vice versa. Moreover, the SIMD measures deprivation on a relative scale within the country, and direct comparison with deprivation in other countries is not possible.

The cause of death could not be determined in one third of the deaths in this study. Therefore, the analyses involving cardiovascular mortality should be interpreted with caution. According to existing data [44], a higher proportion should be expected to die from a cardiovascular cause than the 26.3 % (of the classified death cases) observed in the present study. Many of the unclassified death events in our study were out-of-hospital deaths, probably not infrequently sudden cardiac deaths.

Important covariates were lacking. Tobacco use is a strong predictor of cardiovascular and all-cause mortality [45]. To our knowledge, there is no data suggesting that smoking confound the association between phosphate and outcome, but the association between area deprivation and mortality may have been weakened with adjustment for tobacco use. We also lacked information about use of medication, including antihypertensive drugs, phosphate binders and active vitamin D.

## Conclusions

In summary, we have demonstrated that serum phosphate and area multiple deprivation index were independent predictors of all-cause mortality in a large Scottish cohort of first time attendants to an outpatient renal clinic. The results support similar findings in previous studies. However, despite a higher serum phosphate in the most deprived, deprivation index did not alter the association between phosphate and mortality and vice versa, and there was no interaction between deprivation index and phosphate. Our data support a strategy aimed at lowering serum phosphate in all CKD patients, regardless of socioeconomic status.

## Abbreviations

CKD: Chronic kidney disease
eGFR: Estimated glomerular filtration rate
RRT: Renal replacement therapy
CKD-EPI: Chronic kidney disease epidemiology collaboration
PCR: Protein-creatinine ratio
SIMD: Scottish index of multiple deprivation
HR: Hazard ratio
95 % CI: 95 % Confidence interval
